# College of Health and Health Care Disparities: The Effect of Social and Environmental Factors on Individual and Population Health

**DOI:** 10.3390/ijerph110707492

**Published:** 2014-07-21

**Authors:** Billy Thomas

**Affiliations:** Medicine Center for Diversity Affairs, Department of Neonatology, University of Arkansas for Medical Sciences, 4301 W. Markham St., Mail Slot 625, Little Rock, AR 72205, USA; E-Mail: thomasbillyr@uams.edu

**Keywords:** social determinants of health, health disparities, diversity and inclusion, health care workforce, cultural competency, population health, health literacy, academic pipeline, educational attainment, built environment

## Abstract

Recently the existence and prevalence of health and health care disparities has increased with accompanying research showing that minorities (African Americans, Hispanics/Latinos, Native Americans, and Pacific Islanders) are disproportionately affected resulting in poorer health outcomes compared to non-minority populations (whites). This is due to multiple factors including and most importantly the social determinants of health which includes lower levels of education, overall lower socioeconomic status, inadequate and unsafe housing, and living in close proximity to environmental hazards; all contributing to poor health. Given the ever widening gap in health and health care disparities, the growing number of individuals living at or below the poverty level, the low number of college graduates and the growing shortage of health care professionals (especially minority) the goals of this paper are to: (1) Define diversity and inclusion as interdependent entities. (2) Review the health care system as it relates to barriers/problems within the system resulting in the unequal distribution of quality health care. (3) Examine institutional and global benefits of increasing diversity in research. (4) Provide recommendations on institutional culture change and developing a diverse culturally competent healthcare workforce.

## 1. Introduction

According to the 2012 census, the U.S. population is now greater than 313 million (a 9.7% increase over the past decade) with individuals under 18 representing the most diverse and rapidly growing segment of the population; about 24%. It is predicted that by 2020, no racial or ethnic group will comprise a majority; already, minorities make up about 46% of the under-18 population and about half of all births [1].

Minorities make up 31% of the U.S. population. Individuals of Hispanic or Latino origin make up the largest segment at 16.2% [1]. As the population grows, this young, diverse group of mostly underserved minority individuals will continue to struggle with an educational system that, in many cases, lacks the infrastructure to support their unique needs.

It has been well documented that educational level, socioeconomic status, and health status are all directly linked, are key components of our social structure, and play a major role in how we progress as a society. In 2005, the mortality rate for U.S. adults, ages 25–69, with some education beyond high school was 206/100,000. For those with only a high school education, the rate more than doubled, and tripled for those with less than a high school education; dramatically illustrating the direct correlation between educational attainment, good health, and longevity [2]. Perhaps the most powerful determinant of health over the life- course is educational attainment. How far we go in school will largely determine our income, the neighborhoods in which we live (the built environment), and our access to health care and basic information on healthy lifestyles, and other resources critical for good health. In the US, high school graduation rates are high, with 85.4% of individuals over age 25 earning a diploma. However, only 28.2% of those over 25 have a bachelor’s degree [1]. In addition 14.3% of US citizens live below the federal poverty level [1]. As a result the rapidly growing U.S. population is becoming more diverse, with lower levels of educational attainment and increasing poverty levels. When these factors are combined, the result is poor individual and population health. 

Part of the responsibility of the health care system is to identify those factors (social determinants of health) that negatively influence health within underserved communities and to educate the health care workforce, community, and policymakers about the direct, and in many cases very negative, impact these factors have on overall health, quality of life, and life expectancy. 

Over the past two to three decades the existence and prevalence of health and health care disparities has increased with accompanying research showing that minorities (African Americans, Hispanics/Latinos, Native Americans, and Pacific Islanders) are disproportionately affected resulting in poorer health outcomes compared to non-minority populations (whites) [3,4].

Minorities have poorer health outcomes as it relates to multiple diseases*—*cardiovascular disease, diabetes, asthma, cancer, and HIV/AIDS. This is due to multiple factors, including and most importantly, the social determinants of health which includes lower levels of education, overall socioeconomic status, inadequate and unsafe housing, and living in close proximity to environmental hazards contributing to poor health [3]. 

The identification of health and health care disparities along with their etiologies is only part of the puzzle. Perhaps a bigger and maybe more important part is the development of a workforce that is equipped to address and take on the mounting health care needs of a rapidly growing diverse population. The need for a diverse, culturally competent health care workforce is of primary importance in identifying, addressing and eventually reducing health and healthcare disparities. Nationally, African-Americans, Native Americans, Hispanics/Latinos, and Mainland Puerto Ricans make up 31% of the U.S. population [1]. Minorities account for only 7.4% of medical school faculty, 8.6% of dental school faculty, and <10% of nursing school faulty [5,6,7]. In turning to minority representation in research, Blacks constitute less than 1.0% of researchers with NIH grants [8,9]. In general, there is a very large gap between minority representation in the general population compared to minority representation in health professions and biomedical and behavioral science. 

Given the ever widening gap in health and health care disparities, the growing number of individuals living at or below the poverty level, low college graduation rates, the growing shortage of health care professionals (specifically minorities and individuals from disadvantaged backgrounds) and the currently slow implementation of the Affordable Health Care Act the development and implementation of a health care system that is inclusive, equitable and specifically addresses health and healthcare disparities is critically important. The goals of this article are to: (1) define diversity, inclusion and cultural competency as interdependent entities along with their independent role and combined effects on both individual and population health and health care [10]; (2) review the health care system as it relates to barriers within the system that have resulted in the uneven distribution of equitable quality care and multiple health disparities; (3) examine the benefits of increasing the diversity of the research workforce resulting in the acceleration of advances in medical and public health research; and (4) provide recommendations that will help move us towards a culturally competent health care system and workforce that will be able to provide equitable quality care for a diverse population and close the health and healthcare disparities gap.

## 2. Methods

### 2.1. Defining Diversity, Inclusion and Cultural Competency and Their Effect on Population Health

Why is it important that we not only maintain, but also increase, our current enrollment and graduation rate of minorities (African American, Native American/Alaskan Native, Hispanic, Asian Hawaiian/Pacific Islander, Mainland Puerto Rican and other Asians) in the biomedical sciences? By mid-century, no racial or ethnic group will comprise a majority nationally. The population will be made up of mostly people of color. The Supreme Court ruling in the Grutter *vs.* Bollinger case involving the University of Michigan Law School in which the court reasoned that as population demographics shift, all college graduates will need to be skilled at engaging with diverse individuals, ideas, and values [11]. Nowhere is this more evident than in health care. The need for a diverse student body is a “compelling interest” for educational institutions and the nation; resulting in improved patient access, quality of care, increased patient involvement in the care plan, increased adherence, and more positive patient perceptions of care*—*all leading to reduced health disparities and improved population health [6,12]; all providing valid reasons for pursuing and promoting diversity in higher education.

At most institutions, consideration of race in the holistic admissions process has been supplanted by socio-economic or educationally disadvantaged status*—*either based on family income or educational attainment or quality. While this approach does effectively broaden opportunity for many minority and majority students, the fact remains that the long history of racial discrimination continues to impact minority and disadvantaged students in ways that hinder their chances of success [13,14]. Complete disregard of race or ethnicity as a legitimate factor to consider in the admissions process may shut out deserving candidates. It comes down to whether we as a society, more specifically the US health care delivery system, believe in the educational benefits of diversity*—*that it enriches the quality of education for everyone (students, staff and faculty); individuals with unique personal differences and life experiences contribute to and are part of the learning community. The outcome is a cadre of health care workers who are more open and wiser about the diversity of patients to whom they provide care. 

In behavioral and biomedical science the inclusion of minorities and individuals from disadvantaged backgrounds not only enriches the educational experiences of all in attendance but it also produces a number of researchers that are more likely to pursue areas of research such as diabetes, hypertension, and strokes; all disproportionately affecting minority and underserved populations. Targeted research whether basic, translation or community-based will move us a long way towards reducing health and healthcare disparities. 

### 2.2. Barriers in the Health Care System Resulting in Uneven Distribution of Equitable Quality Care and Multiple Health Disparities

Health care disparities have multiple causes including those originating in the organization of the health care system, patient-provider interactions, and patient characteristics. Perhaps the most malleable are those associated with patient-provider interactions [15]. Is the provider able to communicate effectively with the patient? Does the provider share some past experiences with the patient? Does the provider possess the necessary degree of understanding, humanism and empathy needed to provide equitable quality care? Special emphasis must be placed on effective communication between patients and providers. As the population rapidly diversifies there are a growing number of patients that are either first or second generation immigrants who use English as a second language and have limited English-language proficiency. This is most prominent in the rapidly growing Hispanic population that currently comprises approximately 17% of the US population and is projected to increase to over 33% by 2060 [1,16]. Consequently, by the end of the period, nearly one in three U.S. residents will be Hispanic, up from about one in six today [16]. In the midst of such a large and rapid population shift language discordance between patients and health care providers becomes an increasingly important problem and may account for a large portion of health disparities [17].

In addition Hispanics are also expected to be overrepresented in the more than 30 million newly insured individuals under the Patient Protection and Affordable Care Act (ACA) [17,18]. Stressing the need for immediate infusion of resources and talents into providing interpreter and language assistance services to a rapidly growing population.

Academic Health Centers (AHC) must place special emphasis on the development of a multi-discipline health care workforce that is composed of health care providers who *receive ongoing education and training in culturally and linguistically appropriate service delivery. In addition AHC must be responsible for ensuring workforce awareness of* existing Culturally and Linguistically Appropriate Services (CLAS) standards as they apply to providing interpreters and other language assistance services to individuals with limited English-language proficiency [19].

#### 2.2.1. The Admissions Process 

Many of these questions (above*—*2.2) can be addressed during the admission process to health professions schools. This applies across disciplines. The Association of American Medical Colleges (AAMC) and five other health associations representing schools of osteopathic medicine, dentistry, nursing, pharmacy and public health jointly created the Interprofessional Education Collaborative (IPEC) [20]. This group identified four inter-professional competencies that health professions students should acquire over the course of their training: values and ethics, understanding roles and responsibilities, interprofessional communication and team work [20]. In its most recent revision of the Medical College Admissions Test (MCAT) the AAMC has added a section that tests the knowledge of concepts from behavioral and social sciences to complement the basic and natural sciences [20,21]. In addition as part of its efforts to apply holistic review to the admissions process the AAMC identified the most desirable interpersonal and intrapersonal competencies for entering medical students. These included service orientation, cultural competency, teamwork, ethical responsibility to self and others, reliability and critical thinking [20]. Current efforts by the AAMC to identify and assess interpersonal and intrapersonal competences include a revision of the American Medical College Application services (AMCAS) to include a “Reflection on Interpersonal and Intrapersonal Competencies” section in which students are prompted to reflect on experiences in which they demonstrate some or all of these competencies. In addition the AAMC is exploring the development of a situational judgment test (SJT) which confronts applicants with written or video scenarios and asks them to indicate how they would react by choosing from a list of responses [22,23].

#### 2.2.2. Patient-Provider Relationships: The Value of Familiarity, Past Experiences and Concordance

It has been show that racially concordant provider-patient relationships improve quality of care, increase patient involvement in the care plan, increase patient adherence, and result in a positive patient perception of care [12]. Most researchers feel that in the long run this will reduce health care disparities and improve population health [12]. This moves us towards the development of a culturally competent health care workforce. Although concordant patient:provider interactions produce a very positive effect on overall quality of care it is unrealistic for us to think that in the midst of a rapidly growing diverse population, an ever increasing shortage of health care providers and a very stagnant pool of applicants that identify as either minority or disadvantaged we will be able to catch up or even keep pace in providing the projected number of minority and disadvantaged health care providers that will be needed in forming the “ideal” concordant patient:provider interactions. Instead cultural competency and inclusion must encompass the entire healthcare workforce in order to provide a diverse and culturally competent workforce that can effectively serve an increasingly diverse population.

### 2.3. The Benefits of a Diverse Research Workforce; Accelerated Advances in Medical and Public Health Research—Closing the Health and Healthcare Disparities Gap

The efficacy of healthcare workforce diversity as a pathway to culturally competent care and improved health outcomes has been well documented [12,24]. For underserved and marginalized populations the benefits are better access to and higher quality of health care. 14 % of Americans live below the poverty line and cannot afford health care and are disproportionately affected by multiple health and health care disparities [1]. In the research arena, researchers who are minority are more likely to focus on problems affecting minority and underserved populations, thus accelerating scientific discovery that benefits underserved populations that are disproportionately affected by health and healthcare disparities [24]. Greater faculty and student diversity in the health professions enriches the educational process for everyone, resulting in a more culturally competent health care workforce [15,24].

For the U.S. health care system to set its sights on a more diverse student body and faculty as a way to improve population health sounds simple and straightforward; however, multiple, interdependent factors are at play. This includes the pre-admission process (preparing and recruiting students) and the admissions process (student selection criteria). In addition—and often overlooked—is the importance of retention of minority students, residents, fellows and junior faculty. 

### 2.4. The Benefits of a Diverse Healthcare Workforce—Recruitment and Retention of Minority and Disadvantaged Students and Faculty

#### 2.4.1. Pre-Admissions—Increasing the Pool of Qualified Applicants

Underrepresented racial and ethnic minorities are disproportionately represented among those lacking a postsecondary education. This may be due to the lack of or shortage of educational materials, services, or access that in many cases are available to middle and upper class families*—*books in the home, access to quality pre-K programs, a parent who can serve as a role model and guide for their child’s educational advancement, and well-resourced schools and after-school and summer programs*—*all of which profoundly contribute to success at every point along the academic pipeline (K-16) [25,26,27].

As a result in Arkansas, as it is nationally, the number of the state’s college graduates in biology, biomedical science or the physical sciences – the majors most often leading to a professional degree in the health sciences*—*is small. In 2012 in Arkansas, a total of 2234 students graduated with a bachelor’s degree in the STEM areas; only 15% (350) were minorities [28]. Nationally, whites receive a bachelor’s degree in the biological sciences 2.6 times more often than underrepresented minorities, further contributing to the very small number (percentage) of minority students in the national applicant pool and who are in a position to pursue a graduate or post-graduate degree [29] in the STEM areas.

#### 2.4.2. Recruitment and Retention of Minority Students 

Critical to recruiting and retaining minority students and faculty is the presence of a “critical mass” of minorities, resulting in the generation of an institutional climate that is inviting, nurturing, supportive and inclusive [6,7,30,31]. Without it, it is difficult to convince minority students or faculty that they are valued and supported. Beyond the admissions process there must be greater investment in student support services, such as mentoring, tutoring, student wellness, and “an early warning system” to identify and help students in academic distress [27].

#### 2.4.3. Recruitment and Retention of Minority faculty development—A “Grow Your Own” Approach to Diversity

Growing your own is not a new concept, but it has never been truer. A lack of minority faculty creates an environment that is both uninviting and, in many cases, a deterrent to recruiting and retaining minority students and faculty [6].

Before minority faculty become faculty, they are students. In the case of medical school, if there are only 2600 minority medical students graduating each year in the U.S. [32] and the number is relatively flat, then how do we approach the problem from the perspective of improving population health through structural diversity? Simply shifting students from state to state will not improve national health. Each institution and state must be aware of and avoid the “zero sum effect.” If minority students and faculty are disproportionately distributed between states and institutions, the overall health of the nation suffers. An ideal goal is an equal distribution of minority health care workers in each state in accordance with each state’s demographics, moving towards population parity, improving access and quality of health care, and a reduction in health disparities.

Strengthening programs that nurture the development of students, residents, and postgraduate fellows from diverse backgrounds will result in a gradual increase in the number of minority faculty. Retention efforts must be direct and focused. 

## 3. Discussion

Over the past decade there have been two seminal publications that have provided solid direction and served as a template for organizational and institutional changes that directly address and serve to reduce health and health care disparities. The Institute of Medicine Report*—*Unequal Treatment [15]*—*showed multiple areas in which there are racial and ethnic disparities in the delivery of health care; minorities were referred less for cardiac cath, received less pain medication for fractures, received less surgical treatment of lung cancer, received fewer referrals for renal transplant, received fewer referrals for congestive heart failure and pneumonia, and received fewer major procedures for myocardial infarct (in the elderly) [15]. The report concluded that the healthcare workforce and its ability to deliver quality care for racial and ethnic minorities can be improved substantially by increasing the proportion of underrepresented U.S. racial and ethnic minorities among health care professionals [15]. 

The second publication, Missing Persons: Minorities in the Health Professions, a report by the Sullivan Commission, also points out the key role of diversity in the health care workforce and as a pivotal factor in addressing and reducing health and health care disparities [33]. In summary the Commission states that in order to achieve true equality of high-quality care for the entire population, health care must be provided by a well-trained, qualified, and culturally competent health professions workforce that mirrors the diversity of the population it serves [33].

Both publications provide a very clear and accurate description of a mounting and perplexing problem that severely hampers our current health care system; creating health and health care disparities that ultimately result in poor individual and population health. In addition both publications offer very practical and attainable recommendations that address the problem with the intent of closing the health and health care disparities gap and moving us toward a system of equitable quality care for all*—*good population health.

Health and health care disparities exist across multiple chronic, acute and preventable disease processes including diabetes, cancer, cardiovascular disease, HIV/AIDS, and obesity [4,12,15]. In addition, there are multiple factors that contribute to the disparities in each health care area resulting in a very complex and multifaceted problem. Prominent among these factors are the lack of institutional diversity, inclusion and cultural competency encompassing the entire health care workforce*—*institutional employees, medical staff, students and faculty. All play a major role in developing and sustaining an institutional climate that is inclusive and supportive of all members.

As recently defined by the Group on Diversity and Inclusion of the Association of American Medical Colleges (AAMC) *diversity*, is a core value embodying inclusiveness, mutual respect, and multiple perspectives; resulting in health equity while taking into account individual differences such as socioeconomic status, race, ethnicity, language, nationality, sex, gender identity, sexual orientation, religion, geography, disability, and age [10]. *Inclusion* is a core element to successfully achieving and sustaining diversity. Through professional development, education, policy, and practice inclusion creates a climate that fosters belonging, respect, and value for all and encourages engagement and connection throughout the institution and community [10].

Cultural competency is defined as a set of values, behaviors, attitudes, and practices within a system, organization, program or among individuals which enables them to work effectively cross culturally in providing high-quality care to patients from diverse socio-cultural backgrounds [34]. As outlined by Wells [35] becoming culturally competent is a continuous and longitudinal process that is composed of a cognitive phase and an affective phase. Each phase is composed of three stages beginning with cultural incompetence (a lack of knowledge) in the cognitive stage and ending with cultural proficiency (indicating a mastery of the cognitive and affective phases of cultural development) in the affective phase. It can be argued that we never truly become culturally proficiency but we vacillate between various stages in response to given environmental stimuli and circumstances [35]. Cultural competency extends well beyond the race, ethnic background or gender of a health care provider, but rather is an accumulation and demonstration of knowledge, skills, and attitudes that every health care provider—regardless of training or degrees—should aspire to attain [35,36].

The concepts of cultural competency and inclusion should be integrated throughout the entire U.S. educational system starting at the postsecondary educational level, extending into professional schools and postgraduate training, and beyond as part of a “lifelong learning process” as health care providers seek to deliver equitable quality care in a culturally competent fashion [10,36] 

Each institution and state must move toward self-sufficiency as it deals with the challenges associated with the provision of health care to an increasingly diverse population. What has been touched on in this paper are some of the efforts unfolding on a national level and, more specifically, at the University of Arkansas for Medical Sciences (UAMS) to identify and address both system and patient:provider issues, such as the lack of cultural competency and diversity and inclusion in addition to healthcare workforce issues that result in health and healthcare disparities [12,15,35].

## 4. Recommendations and Conclusions

### 4.1. Continued Support and Development of Summer Academic Enrichment Programs

This will facilitate the identification and nurturing of students that have an interest in and are willing to make the effort to pursue careers in the STEM areas. Many students from disadvantaged backgrounds benefit enormously from their participation in science based programs. Besides providing exposure to solid science and math programs summer academic enrichment programs provide a continuing level of engagement and support for students during the summer months; reducing the negative effects of time spent out-of-school (summers, weekends) during which students tend to fall behind. Time spent out-of-school widens the educational gap [37,38,39,40]. This is more prominent in low income underserved communities. The result is a very high attrition rate especially at the middle school level and a very low college going and completion rate [37,38]. Levels of educational attainment directly correlate with housing, poverty, ability to afford health care, health literacy and in the end individual and population health [2].

### 4.2. An Increase in the Number of URM Faculty at Health Professional Schools

Nationally minorities account for only 7.4% of medical school faculty (with 20% located at Historically Black Colleges), 8.6% of dental school faculty, and <10% of nursing school faulty [5,6,41]. In turning to minority representation in research, Blacks constitute less than 1.0% of researchers with NIH grants [8,9]. A crucial component to our success in reducing health and health care disparities is the recruitment and retention of minorities across disciplines in positions of leadership, health system design, and research.

Currently at UAMS minority faculty makeup 6.8% of UAMS faculty*—*African American or Black (3.8%), Hispanic (2.5%), Native Hawaiian or Other Pacific Islander (0.07%) and American Indian/Alaskan Native (0.4%). Underrepresented minorities make up 23% of the state population. So the very low number of URM’s that are part of the UAMS faculty falls short of population parity. This lack of visible URM faculty creates an environment that is less welcoming for recruiting URM students and faculty. The Minority Recruitment and Retention Committee (MRRC) which is composed of faculty and student representatives from each of the five (5) colleges on the UAMS campus has as a primary goal the identification and recruitment of URM students and faculty. Recently the committee submitted a list of recommendations to the Chancellor and other top level administrators. Recommendations include the following: *development of standardize campus-wide procedure for monitoring the recruitment and retention of minority faculty; centralize monitoring of recruitment and retention efforts in the Center for Diversity Affairs and Human Resources (HR); incentivize efforts on the part of administrators and faculty to improve faculty diversity; develop a set of best practices to improve faculty diversity; increase recruitment efforts—targeted hiring, cluster hires, opportunity hires; assign diversity advocate to search committees; incentivize diversity activity at the department level; as part of the promotion and tenure process increase protected time for URM researchers who are more likely to pursue community-based research; and a Diversity Award (student and faculty) has been established.* Recommendations have been made part of the strategic plan of the Center for Diversity Affairs with plans for implementation over the next two (2) years.

### 4.3. Collection of Demographic Data (Racial and Ethnic) in Clinical or Health Services Research

It is well documented that the lack of minority participation in multiple clinical trials result in the collection of data and study outcomes that may not be valid when applied to the treatment of minorities and other marginalized populations [42,43,44,45,46].

The lack of minority participation in clinical trial is due to multiple factors including mistrust of government and research institutions, low health literacy, beliefs about limited benefits from results, and lack of information about trials [45,46]. One approach to increasing minority participation is through improved health literacy. Improvement in health literacy of the entire population will allow us to not only improve individual and population health but will also highlight the value of participation in clinical studies. Health literacy is defined as the degree to which individuals have the capacity to obtain, process, and understand basic health information and services needed to make appropriate health decisions [47]. The consequences of inadequate health literacy include poorer health status, lack of medical care knowledge, impaired comprehension of medical information, lack of knowledge about medical conditions, lack of understanding and use of preventive services, poorer self-reported health, poorer compliance rates with treatment modalities, increased hospitalization, and increased health cost [48]. All fall under the umbrella of preventative services. Improved health literacy is key to improving individual and population awareness of healthy choices (life style modifications*—*diet, exercise, no smoking, reduced alcohol consumption), the value of preventative primary care services and in the end overall impact of health literacy on individual and population health. This can be done at the local, state and national level through community-based educational campaigns that must include on site community involvement and a series of lectures and workshops that are developed in collaboration with community leaders in response to community needs/requests, and open to the public. 

### 4.4. Research That Will Identify Root Causes and Strategies to Eliminate Racial and Ethnic Disparities

Future research must include an increasing number of minority faculty. Efforts to increase minority faculty start at the K-16 level and extends into and beyond professional school. Faculty recruitment and retention is crucial to institutional and environmental change that will promote recruitment and retention of students and the development of a “critical mass” of minority researchers [7,30,31].

Many researchers develop an interest in and pursue careers in fields of research in which they have past professional or personal experience. Many minority and disadvantaged researchers pursue careers in cardiovascular disease, diabetes, obesity, HIV/AIDS, and stroke; all health disparities areas which disproportionately affect minority and underserved populations [4,6,12]. An increase in the number of minority researchers that are actively involved in research will help close the disparities gap. This should include continued NIH support and formal training in grant writing and the grant review process at the institutional level (how to respond to targeted RFAs, developing an awareness and working knowledge about the grant review process—evaluation and scoring). Additional institutional efforts should include development of Leadership Programs that specifically target minority faculty.

The NIH has several program grants through multiple institutes that target and support students and faculty researchers from minority, disadvantaged and underserved populations. Currently the Center for Diversity Affairs (CDA) in conjunction with the UAMS Graduate School has two NIH training grants targeting minority, underrepresented (UR) and disadvantaged students at the undergraduate and graduate level. The Initiative to Maximize Student Development (IMSD) targets UR and disadvantaged students pursuing a PhD in behavioral and biomedical sciences and is funded by the National Institute for General Medical Sciences (NIGMS). Since the institution of the IMSD program we have more than doubled our enrollment of UR and disadvantaged student in our Graduate School. The Summer Undergraduate Research Program (SURP) provides support for mentored summer research targeting minority and disadvantaged students at the college level. Both are excellent programs and have had a significant institutional impact in helping us to diversify our student body not only in the graduate school but also across colleges and disciples.

### 4.5. Development of Sustainable Community-Based Partnerships to Evolve and Expand Potential for Health Disparities Reduction Research, Practices and Policy Formation

Recently the Center for Diversity Affairs submitted a proposal to the National Institute on Minority Health and Health Disparities (NIMHD) to support a conference and a series of lectures and workshops on health and health disparities*—the Jocelyn Elders Lecture Series*. The overall objective of the conference and lecture series is to expose and educate the state and regional health care workforce (primary care providers; across disciplines and including students, faculty and staff) and the community to the ever increasing problem of health and health care disparities; ultimately promoting a reduction in health and health care disparities resulting in improved individual and population health. Community partners include the Arkansas Medical Dental and Pharmaceutical Association (AMDPA) a 120 year organization primarily composed of local and regional minority physicians, dentist, and pharmacists; the Better Community Developers a community-based organization with a focus on low-income, underserved, disadvantaged families and children at-risk; and Tri County Rural Health Network whose primary focus is connecting elderly and adult disabled citizens to home and community-based services. These along with other community partnerships are currently in place; program activities are supported by institutional funds and beginning to make a significant difference in underserved communities surrounding the UAMS campus. In addition the UAMS 12th Street Health and Wellness Center is an interprofessional center that provides preventative and minor “sick” care services to uninsured individuals from local underserved neighborhoods. The Center is student lead with UAMS faculty and local community health care providers serving as preceptors. Students from across disciplines (pharmacy, medicine, nursing, allied health, public health and the graduate school) work as a team in providing information on healthy living, preventive care and referral for chronic care follow-up.

### 4.6. Institutional Support of Language Assistance Services

AHC must place special emphasis on the development of a multi-discipline health care workforce that is composed of health care providers who receive ongoing education and training in culturally and linguistically appropriate service delivery. AHC must be responsible for ensuring workforce awareness of existing CLAS standards as they apply to providing interpreters and other language assistance services to individuals with limited English-language proficiency [19]. This in addition to the aggressive recruitment and retention of Hispanic students across disciples will result in a health care workforce that may be best suited and most likely to delivery equitable quality care to a growing population of Hispanic patients.

### 4.7. A Reduction in Health Disparities through the Identification, and Reduction or Elimination of Population Specific Social Determinates of Health

This takes on various aspects of a social and political nature. It is a combination of actions which include evaluating and perhaps restructuring the current US K-16 educational system that will promote and allow an increase in educational attainment levels; effectively reducing poverty levels and promoting the development of a pool of competitive minority and disadvantaged students that apply to and matriculate into health professions schools; ultimately adding to the pool of health care providers and producing a cadre of minority physicians and researchers. A reduction of health disparities must be a key component of the US health care system. As we approach the issue of increasing the number of underrepresented minorities and individuals from disadvantaged backgrounds who apply to professional school perhaps a broader and more complex approach maybe the institution of community services and community programs that identify and serve to relieve or avert many of the toxic stressors that are so common in underserved neighborhoods. AHC are major forces in economic development—creating jobs, developing innovative ways to improve and save lives, and providing care to the community. Many often rank among the largest employers in their city, region, and/or state and in most cases are the largest annual providers of uncompensated patient care [4,49].

In addition many AHC are in close proximity to underserved communities; either surrounding or bordering them [4]. AHC’s must reach out to surrounding communities, establish a real physical presence and develop partnerships that will result in very early interventions that may reduce environmental stressors. Many minority and disadvantaged students come from the same communities that surround many AHC’s and many are continually exposed to toxic stressors that may hinder mental and behavioral development resulting in poor school performance, high attrition rates and low level of educational attainment [50]. Tangible widespread community involvement is a long term investment that academic medicine and the US health care system must be willing to make; otherwise our goal of diversifying the health care workforce and reducing health and health care disparities may not be realized.

## Notes Added in Proof

“In addition as part of its efforts to apply holistic review to the admissions process the AAMC identified the most desirable interpersonal and intrapersonal competencies for entering medical students. These included service orientation, cultural competency, teamwork, ethical responsibility to self and others, reliability and critical thinking [20]. Current efforts by the AAMC to identify and assess interpersonal and intrapersonal competences include a revision of the American Medical College Application services (AMCAS) to include a “Reflection on Interpersonal and Intrapersonal Competencies” section in which students are prompted to reflect on experiences in which they demonstrate some or all of these competencies. In addition the AAMC is exploring the development of a situational judgment test (SJT) which confronts applicants with written or video scenarios and asks them to indicate how they would react by choosing from a list of responses [22,23].” was added on page 5, lines 11–20.
